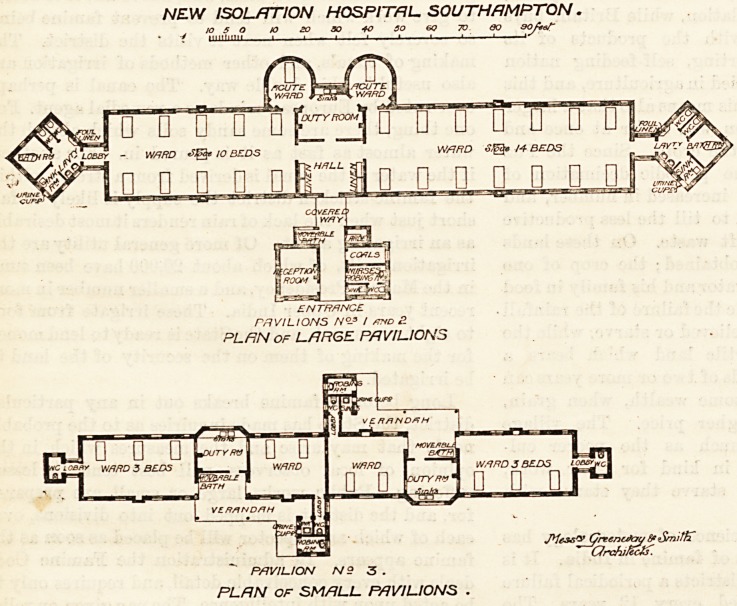# Hospital Construction

**Published:** 1900-09-01

**Authors:** 


### The Institutional Workshop.

### HOSPITAL CONSTRUCTION.

### THE NEW ISOLATION HOSPITAL FOR SOUTHAMPTON.

The passing of the Infectious Disease Notification Act of 1889 has had the effect of stimulating the authorities to provide suitable hospitals in which to treat these diseases, and few towns are now better provided for in this respect than Southampton. As late as 1892 the hospital consisted "of a private house, incapable of receiving more than one variety of infectious disease at the same time;" and in 1893 temporary accommodation was secured by running up an iron hospital near the West Quay. In that year small-pox broke out; the hospital was occupied by scarlet fever cases; suitable premises could not be secured, and over £4,000 was spent in coping with the epidemic. The Health Committee say that this experience was not thrown away, and a port sanatorium was provided for the treatment of small-pox. The committee take a most enlightened view of their duties, and they say that the cost of maintaining the floating hospital "must be considered an insurance; for it is impossible to estimate the value of successfully combating an outbreak." We have gone somewhat fully into these preliminaries, because there must be many districts in the same position as Southampton formerly was, and if these realise what the Health Committee have gone through, and how successfully their difficulties have been surmounted, such knowledge may form an inducement for those unprovided boroughs to act likewise.

NEW ISOLATION HOSPITAL SOUTHAMPTON.

PAVILIONS Nos 1 and 2

PLAN of LARGE PAVILIONS

PAVILION No 3.

PLAN of SMALL PAVILIONS.

The Southampton Isolation Hospital is situated on ten acres of high ground near Shirley. The entrance is placed at the south-east corner of the land in Mousehole Lane. The subsoil is red gravel.

There is an "out-bathing station" on the main road. This contains two undressing-rooms, two bath-rooms, one dressing-room, and a waiting-room. By these means a patient on discharge is conveyed from the hospital to the undressing-room on the north side of the block, and after bathing will dress and be handed over to his friends at the waitingroom on the south side of the block.

The administration block is placed to the west of the main entrance. It contains on the ground floor all the usual rooms and offices, and all these are well arranged. The first and second floors contain 21 bedrooms for the staff, with bath-rooms, lavatories, &c.

At present only four pavilions have been erected, but there is ample space for future extension. Pavilions Nos. I. and II. are identical. Each is designed for 26 beds. The long axis of the building runs slightly east of north and west of south, and the pavilion is unequally divided into two parts by the hall and the duty-room. On one hand is a dormitory for 14 beds, and on the other is one for 10 beds; and each dormitory has projecting from its corner a single-bedde room. This single-bedded room is semi-circular, a plan which permits of easy ventilation inside it, and which obstructs less light and air from the adjoining dormitories and duty-room than would any other design. A door leads from the du room to each single-bedded room, and from the latter to the dormitory.

At the end of each dormitory is the sanitary block, carefully cut off by a ventilating passage, and containing bath-room, lavatory, sinks, and closets. Unquestionably the most has been made of this space, and the plan (which we do not remember having seen before) of putting the block on at an obtuse angle to the ventilating passage has the effect of securing more light and air than the usual form. A covered way leads from the hall to a small block containing the reception-room, nurses' robing-room, coal stores, movable bath, &c. The single-bedded rooms have open fireplaces, and the wards are heated by Doulton-ware stoves. This is an admirable system so far as the production of warmth goes; but no ward should be exclusively heated in this or in any other "system." Open fireplaces should invariably be present. Where everything seems to have been so carefully thought out, and so well executed, it is a pity that this point was overlooked. There are fresh-air inlets.

Pavilion III. consists of four wards arranged in pairs, each pair being separated by a duty-room. Two of the wards contain three beds each, and two contain two beds each; and from each of the smaller wards runs a passage connecting it with a block containing a robing-room and other offices. A verandah connects the wards with this block; and this arrangement, which is very well carried out, permits two diseases to be isolated in the same block without fear of communication. This pavilion is intended for typhoid fever and diphtheria.

Pavilion IV. is very similar to Pavilion III.; but no ward has more than two beds, and these small wards are intended for private patients and for the isolation of doubtful cases. Pavilions III. and IV. are heated by open fireplaces and radiators. The closets are placed at the extreme ends of the blocks, but bath-rooms are not provided, as movable baths will be used; but we are of opinion that fixed baths would have been found very useful during the convalescence of patients. The walls are finished with Robinson's cement, and the floors are laid with pitch pine. The laundry is placed westwards of the administration block, and is fitted with Washington Lyons' disinfector, and everything necessary for rapidly and effectually washing and drying the linen of a fever hospital. The drainage is carried to the extreme south-east corner of the estate in earthenware pipes, and there joins the main sewer. The buildings are lighted by electricity. The cubic space allowed is 2,000 ft. for scarlet fever cases, and 2,500 for typhoid and diphtheria. Calculated on this basis the hospital is sufficient for 72 beds. The architects were Messrs. Greenaway and Smith, of Queen Anne's Gate, Westminster; the contractors were Messrs. Jenkins and Son, Southampton; for engineering, Messrs. Moorwood and Co.; for heating, Messrs. Russell and Co. The cost of the building was £22,827; and £7,173 is expected to cover the cost of heating, lighting, furniture, and contingencies.

## Figures and Tables

**Figure f1:**